# Forecasting hospital demand in metropolitan areas during the current COVID-19 pandemic and estimates of lockdown-induced 2nd waves

**DOI:** 10.1371/journal.pone.0245669

**Published:** 2021-01-22

**Authors:** Marcos A. Capistran, Antonio Capella, J. Andrés Christen

**Affiliations:** 1 Centro de Investigación en Matemáticas, CIMAT, Guanajuato, Guanajuato, Mexico; 2 Instituto de Matemáticas, UNAM, Circuito Exterior, CU, CDMX, Mexico; University of Surrey, School of Veterinary Medicine, UNITED KINGDOM

## Abstract

We present a forecasting model aim to predict hospital occupancy in metropolitan areas during the current COVID-19 pandemic. Our SEIRD type model features asymptomatic and symptomatic infections with detailed hospital dynamics. We model explicitly branching probabilities and non-exponential residence times in each latent and infected compartments. Using both hospital admittance confirmed cases and deaths, we infer the contact rate and the initial conditions of the dynamical system, considering breakpoints to model lockdown interventions and the increase in effective population size due to lockdown relaxation. The latter features let us model lockdown-induced 2nd waves. Our Bayesian approach allows us to produce timely probabilistic forecasts of hospital demand. We have applied the model to analyze more than 70 metropolitan areas and 32 states in Mexico.

## Introduction

The ongoing COVID-19 pandemic has posed a major challenge to public health systems in many countries with the imminent risk of saturated hospitals and patients not receiving proper medical care. Although the scientific community and public health authorities had insight regarding the risks and preparedness measures required at the onset of a zoonotic pandemic, our knowledge of the fatality and spread rates of COVID-19 remains limited [1–4]. In terms of disease handling, two leading issues determining the current situation are the lack of pharmaceutical treatment and our inability to estimate the extent of the asymptomatic infection of COVID-19 [5–7].

Under current circumstances, control measures reduce new infections by limiting the number of contacts through mitigation and suppression [1]. Mitigation includes social distancing, testing, tracing, and isolating infected individuals, while suppression imposes temporary cancellation of non-essential activities. Mitigation and suppression pose a burden on the economy while affecting more severely individuals living in low-income conditions and challenging populations’ capacity to comply with control measures. As lockdown measures are eased, more people become in contact with the outbreak, and there is a risk of induced 2nd waves that may increase healthcare system pressure.

Data-driven epidemiological models are built out of the necessity of making forecasts. There are many lessons learned on emergency preparedness and epidemic surveillance from previous pandemic events: AH1N1 influenza [8], MERS [9], SARS [10], Zika [11], Ebola [12], etcetera. However, surveillance data during a pandemic event often suffer from serious deficiencies such as incompleteness and backlogs. Another critical issue is the design of data acquisition, taking into account geographical granularity [13]. Epidemic surveillance of COVID-19 is no different since there is an unknown percentage of asymptomatic infections, and susceptibility is related to economic vulnerability.

Undoubtedly, one key task during the early pandemic response efforts is using epidemiological records and mathematical and statistical modeling to forecast excess hospital care demand with formal quantified uncertainty.

In this paper, we pose a compartmental SEIRD model that considers both asymptomatic and symptomatic infection, including hospital dynamics. We model the residence time in each latent and infected compartments explicitly [14, 15], and we use records of daily confirmed cases and deaths to pose a statistical model that accounts for data overdispersion [16, 17]. Furthermore, we use Bayesian inference to estimate the initial state of the governing equations, the contact rate, and a proxy of the population size to make probabilistic forecasts of the required hospital beds, including the number of intensive care units. The model output has been used by Mexican public health authorities to assist decision making during the COVID-19 pandemic in more than 70 metropolitan areas and the country’s 32 states.

### Contributions and limitations

- We developed a model to produce accurate midterm (several weeks) probabilistic forecast of COVID-19 hospital pressure, namely hospital beds and respiratory support or mechanical ventilation demands, using confirmed records of cases at hospital admittance and deaths.
- Our model accounts for policy changes in control measures, such as school closures [18] and lockdowns, as breakpoints in the transmission rates.
- Assuming a given fraction of asymptomatic individuals, we infer changes in the transmission rate and the effective population size before and after a given lockdown–relaxation day.
- Inferred changes in effective population size allows us to produce a forecast of lockdown-induced 2nd waves.

Since asymptomatic infection is not fully understood so far [19], the fraction of asymptomatic individuals is yet unknown. Therefore:

- The effective population size is only a proxy, and its absolute value is not meaningful, but only its relative value before and after a relaxation day.
- Without serological studies in the open population—ideally after an outbreak–it is impossible to forecast the population fraction that will be in contact with the virus by the end of the current outbreak.
- At this point, our model does not address next pandemic outbreaks beyond lockdown-induced 2nd waves.
- Finally, although the model does not account explicitly for biases due to behavioral changes [20, 21], population clustering and super spreading events [22], we argue that our approach to lockdowns and relaxation events is a proxy model of these more general events.

### Related work

There are many modeling efforts aimed at forecasting the number of cases, deaths and hospital occupancy during the ongoing COVID-19 pandemic [23–28]. Broadly speaking, models are informed with evolving information about COVID-19 cases, clinical description of the patient residence time in each compartment, fraction of cases per age group, number of deaths, hospital bed occupancy, etc. Columbia University metapopulation SEIR model [23] forecasts are based on assumptions relating an effective contact rate with population density at a metropolitan area and social distancing policies. The COVID Act Now model [24] forecasts the effective reproduction number *R*_*t*_ and the fraction of infections requiring hospitalization using the Bayesian paradigm to fit a SEIR model to cases, hospitalization, death, and recovery counts. The Imperial College response team mathematical model [25] uses an unweighted ensemble of four models to produce forecasts of the number of deaths in the week ahead for each country with active transmission. The IHME model [26] combines a mechanistic model of transmission with curve fitting to forecast the number of infections and deaths. Moghadas *et al*. [27] pose a mechanistic model parametrized with demographic data to project hospital utilization in the United States during the COVID-19 pandemic. The main goal of Moghadas *et al*. is to estimate hospital pressure throughout.

Other COVID-19 models have been used to explore exit strategies [29, 30], the role of recovered individuals as human shields [31], digital contact tracing [32], break points in the contact rate to account for changes in suppression and mitigation policies [18] and lockdown-induced 2nd COVID waves [33] under the assumption that population is temporally geographically isolated.

## Materials and methods

“Models should not be presented as scientific truth” [34]. Indeed, models are tools intended to serve a specific purpose, evaluate or forecast particular aspects of phenomena and ideally should be developed following the processes of predictive science [35]. Namely, identify the quantities of interest (QoI), verify the computational and mathematical approximation error, including their implication in the estimation of QoI, and calibrate the parameters to adjust the model in light of data to bring it closer to experimental observation. When considering uncertainty, Bayesian inference may be used to calibrate some key model features given data. Finally, a validation process must be used to build confidence in the accuracy of the QoI predictions. Our model is built out of three interrelated components; a law for dynamics, a law for uncertainty, and the choice of parameters.

### Dynamical model

**Fig 1:**
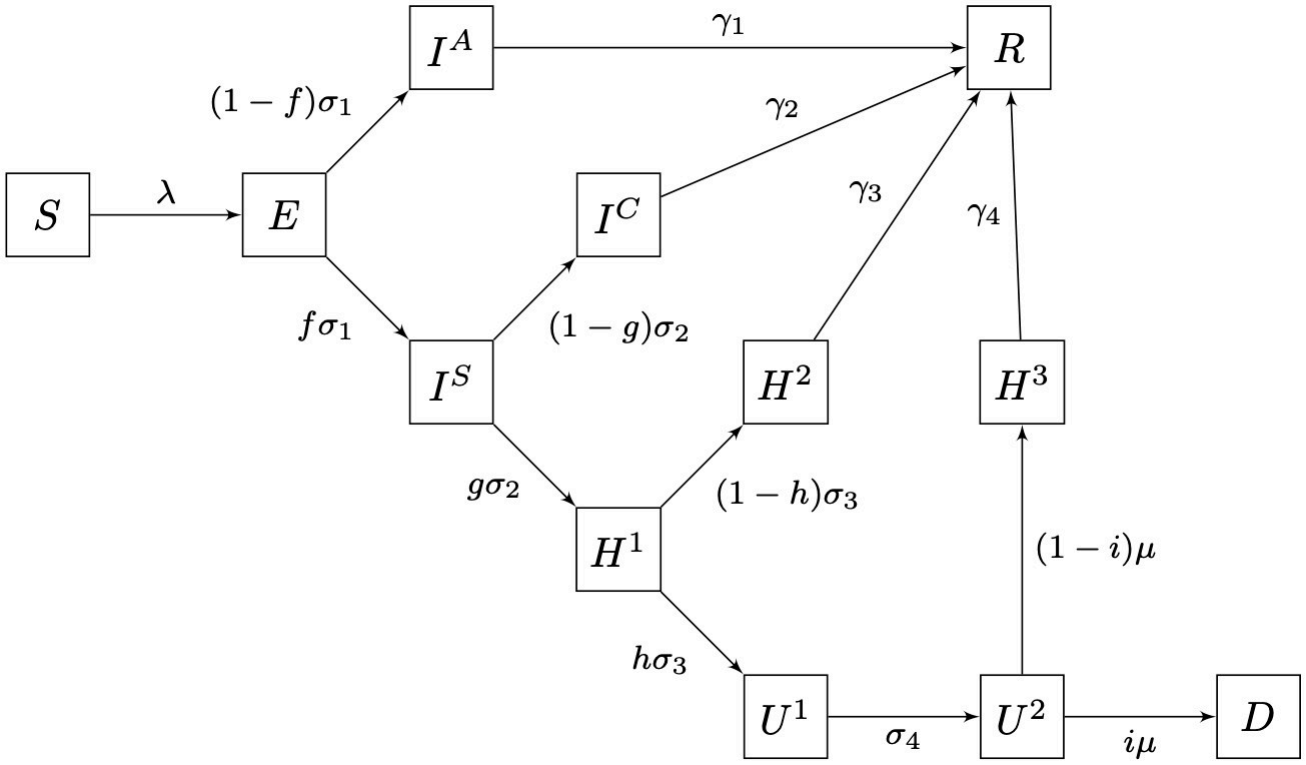
Schematic diagram of the dynamical model. Erlang sub-compartments not shown. For a precise definition of parameters, see S1 File.

As a proxy of hospital pressure, the quantities of interest in our model are the evolving demand of ICU/respiratory–support beds and no-ICU hospital beds. We developed a full compartmental SEIRD model featuring several compartments to describe hospital dynamics (see Fig 1 and S1 File, SM) with sub-compartments to model explicitly residence rates as Erlang distributions [14, 15].

Succinctly our model goes as follows: once the susceptible individuals (*S*) become infected, they remain in the incubation compartment (*E*) for mean time of 1/*σ*_1_ days (i.e. residence rate *σ*_1_). After the incubation period, exposed individuals become infectious and a proportion *f* of them become sufficiently severe symptomatic cases (*I*^*S*^) to approach a hospital, while remaining cases become mild–symptomatic to asymptomatic (*I*^*A*^). The asymptomatic/mild–symptomatic cases remain infectious a mean time of 1/*γ*_1_ days and eventually recover. For the symptomatic cases (*I*^*S*^) we assume that after an average time of 1/*σ*_2_ days a proportion *g* of infected individuals will need hospitalization (*H*^1^), while the rest (*I*^*C*^) will receive ambulatory care, recovering after an average convalescent time of 1/*γ*_2_ days in quarantine. The hospitalized patients (*H*^1^) remain an average time of 1/*σ*_3_ days until a fraction *h* will need assisting respiratory measures or ICU care such as mechanical ventilation (*U*^1^). The remaining fraction 1 − *h* of hospitalized patients (*H*^2^) will recover after 1/*γ*_3_ days in average.

Respiratory-assisted/ICU patients (*U*^1^) remain in that state an average of 1/*σ*_4_ days until they move into the critical state (*U*^2^) with residence time 1/*μ* days until a critical day is reached when a proportion *i* of them will die (*D*) and the remaining proportion 1 − *i* will recover (*H*^3^) after an average period of 1/*γ*_4_ days.

Similar models have been proposed by [2, 31, 32, 36]. For the infection force (λ) we assume that only mild–symptomatic/asymptomatic (*I*^*A*^) and symptomatic (*I*^*S*^) individuals spread the infection, that is

$$\begin{array}{c} \lambda =\frac{\beta_{A}I^{A}+\beta_{S}I^{S}}{N_{eff}}, \end{array}$$

where *β*_*A*_ and *β*_*S*_ are the contact rates of asymptomatic/mild–symptomatic and symptomatic individuals, respectively.

### Parameters and observational model

The model has two kinds of parameters that have to be calibrated or inferred; the ones related to COVID-19 disease and hospitalization dynamics (such as residence times and proportions of individuals that split at each bifurcation of the model) and those associated with the public response to mitigation measures such as the contact rates *β*’s and the effective population size *N*_*eff*_ during the outbreak. Some of these parameters can be estimated from hospital records or found in recent literature or inferred from reported cases and deaths, but some remain mostly unknown. In the latter category, we have *N*_*eff*_ and the fraction 1 − *f* of asymptomatic/mild–symptomatic infections. Reported values of the proportion of asymptomatic/mild–symptomatic infections cases 1 − *f* range from 10% to 75%, and even 95% in children population [6, 7, 37]. The number *N*_*eff*_ is lower than the full population of a metropolitan area and depends on different aspects. Still, it is likely to be a consequence of unequal observance of social distancing public policies among the population that, in turn, yield some clustering effects. As lockdown measures are relaxed, more people become in contact with the outbreak, and *N*_*eff*_ may change. This change is a proxy of the fact that the connectivity between clusters increases, and new paths become open to the virus to colonize the full population. In our model the total number of patients that will visit a hospital is given roughly (bounded) by the product *N*_*eff*_ × *f* and the total number of patients admitted to the hospital is given by *N*_*eff*_ × *f* × *g*, where *g* is the portion of infected persons that need hospitalization.

Since our QoI are concerned with hospital pressure, we choose from the available data two sources of information for the observational model: The registered confirmed COVID-19 patients at hospitals, with or without hospitalization, and deceased patients. Even under an outbreak, these data are reasonably consistent and systematic information on the inflow, (i.e. hospitalized confirmed cases), and the outflow (i.e. deceased), that “hedge” the hospital dynamics.

We remark that our modeling and observation approach neglects hospital saturation. Indeed, limited healthcare capacity would undoubtedly limit the usefulness of our model. Since we care about forecasting hospital pressure, we assume the number of hospital beds and ICU may be increased as needed per metropolitan area to meet the demand.

We have evidence (see S1 File) that given our choice of observation model, the inference of our QoI only depends on the product *N*_*eff*_ × *f* × *g*, not on the value of their factors. The fraction *g* is easy to estimate from hospital records (see S1 File) of admissions and ambulatory patients. Thus we are only required to postulate a value for the product *N*_*eff*_ × *f*. As will be explained later (see also S1 File), there exists a confounding effect related to the product *N*_*eff*_ × *f* that has consequences on the choice of postulated and inferred parameters before and after the outbreak’s peak.

### Lockdowns, relaxation and lockdown-induced 2nd waves

Without vital dynamics, constant parameters, SEIRD-type models predict that the infected population dynamics will have a single outbreak wave. Changes in the transmission rate or secondary lockdown-induced waves arise from exogenous changes in model parameters related to the lockdown onset and offset.

Our model features two types of changes in parameters: lockdown intervention and lockdown relaxation. To model lockdown interventions, a breakpoint is established at which *β* = *β*_1_ before and *β* = *β*_2_ after the intervention day. This creates a non-linear time-dependent *β*(*t*) [18, 38]. Other intervention days may be included in the same fashion by adding more change points and *β* parameters. These additional parameters are then included in the inference.

Assuming that effective population size *N*_*eff*_ and transmission rates are fixed, SEIRD type models converge to the attractor *E* = 0, *I* = 0, i.e., the system models an epidemic that dies out after one single peak. Even for sensible non–constant transmission rates, these kinds of models can only produce single epidemic outbreaks with a limited peak height.

Therefore, in order to be able to estimate secondary outbreak waves after lockdown relaxation measures, one necessarily needs to estimate a different system size, i.e. *N*_*eff*_, before and after a relaxation day. System size increases are related to transmission heterogeneity between social groups and lockdown induced decomposition of socio-geographic networks into connected components. That is clusters of people that are indirectly in contact with each other through direct contacts. As lockdown starts, there is an induced network unreachable between different clusters, and fewer individuals participate in the first outbreak wave. At the end of the lockdown, people previously isolated from the outbreak become susceptible as contacts between different clusters switch back on [33].

Thus, we model relaxation days as changes in both the transmission rate *β* and the effective population size *N*_*eff*_. Our approach here is as follows: we include the new parameter(s) *ω*_*i*_ ∈ (0, 1) and set *N*_*eff*_ = *ω*_*i*_ *N*, where *N* is the total population of the metropolitan area or region under study. Next, we postulate a fixed value of *f* and estimate *ω*_1_ and *ω*_2_ before and after the relaxation day, respectively. As explained above, we model possible changes in population behavior by letting the transmission rate *β* vary before and after a relaxation day. With this procedure, we can estimate *N*_*eff*_ in terms of the *ω*’s. Nevertheless, due to the confounding effect of the product *N*_*eff*_ × *f* = *N* × *ω* × *f*, we are still unable to estimate the actual *N*_*eff*_’s until the real value of *f* is known.

### Setting lockdown and relaxation days

As explained before, we model interventions and relaxation days as discontinuities in *β* and (*β*, *ω*), respectively. We consider a lockdown day on 22 March 2020, where a country-wide lockdown started. We include a second intervention-day in Mexico City to model further local lockdown measures in early April.

Setting up relaxation days is more delicate. In countries with stark income contrasts, the follow-up of lockdown measures is unequal among different social groups. Due to economic differences in social groups, the increase in network connectivity is not necessarily related to a single event or an official policy change. Therefore, besides the official lockdown onset and offset dates, it is necessary to consider differentiated groups’ behavioral changes that modify the outbreak’s evolution. Thus, several relaxation days and a methodology to define them is needed.

There are many choices to establish relaxation days. It is possible to include the number and dates of relaxation-days as part of the inference. However, this results in a complex transdimensional Markov chain Monte Carlo (MCMC) [39] for the analysis of the resulting posterior distribution. We use a self-tuned semi-automatic MCMC algorithm (the t-walk, see Bayesian inference below), and an equivalent transdimensional MCMC is, as yet, not available. The added complexities of this alternative make it impractical and perhaps prohibitive. On the other hand, our more pragmatic approach to be explained next leads to a feasible analysis and satisfactory results. See the S1 File for a more detailed discussion.

Here we consider the selection of relaxation days as a modeling problem. We propose to use the *effective reproduction number* as an auxiliary tool to detect change points in contagion patterns. This procedure seems robust concerning changes in the selected relaxation day (see S4 in S1 File).

The effective reproduction number *R*_*t*_ quantifies the expected number of people infected by a typical contagious person. On the one hand, there exist different possible procedures to estimate *R*_*t*_ from reported cases, e.g., [40]. On the other hand, in compartmental models, *R*_*t*_ reduces to combining model parameters and state variables. For instance, in a simple SIR model [41], *R*_*t*_ = *βS*/(*γN*), where *β* is the infectious rate, *γ*^−1^ measures the duration of the contagious period, and *N* is the population size. When *R*_*t*_ becomes smaller than one, the number of newly infected individuals decreases and indicates that the pandemic wave has reached its peak. In constant parameters SIR models, it can be showed that *S* is a monotonously decreasing function of time, and so is *R*_*t*_. Therefore, a local minimum in the data-estimated *R*_*t*_ most likely represents a structural change in contagion due to groups’ behavioral changes in the real epidemic. This structural change is either because of an increase in the infectious rate or an increase in the system’s size. Here, by a system size increase, we mean that only *S* and *N* increase by a fixed amount among all state variables. Both effects may also be present simultaneously. Another possibility is a change in *γ*^−1^, but it is less likely since it depends on the nature of the disease.

Therefore we introduced the relaxation-days as the times where we have evidence (through local minima in the data-estimated *R*_*t*_ series) of structural changes, see Fig 5. Given the above discussion, we integrate the structural changes into the model as modifications in *β*, *ω*, or both. Notice that an increase in *ω* implies an increase in the system size *N* and the susceptible individuals *S*(*t*). We also notice that the ratio *S*(*t*)/*N* is approximately equal to 1, regardless of the system’s size at the beginning of the outbreak. Therefore, *R*_*t*_, as defined above, is close to *R*_0_ ≔ *β*/*γ*, and any change in contagious is only due to changes in *β*. Not until saturation effects near the outbreak’s peak become evident, we start to have information about the system’s size. This observation reinforces our proposal to consider two kinds of intervention points, one before the peak that modifies *β* and another after the peak where we modify both *β* and *ω*. Our proposal to consider structural changes by monitoring *R*_*t*_ is independent of the particular compartmental model considered. It is an extension that allows us to incorporate some features of the changing socio-geographic network structure into our simple compartmental model—a crucial point for any forecast during a pandemic outbreak.

### Observational model and data

To make our inferences, we use both confirmed cases and deceased counts. In some regions, sub reporting of COVID-19 related deaths may become relevant, especially in places hit by a severe outbreak [42]. Nonetheless, deaths are a more reliable data source to estimate a COVID-19 outbreak, especially in the forecast of hospital demand. The problem here is that the number of confirmed cases depends heavily on local practices, particularly with the intensity of testing, adding a complication if testing intensity has varied due to ambiguous policies. Regarding data from Mexico, patients are tested upon arrival at hospitals with probable COVID-19 symptoms, and limited testing is done elsewhere; accordingly, most confirmed COVID-19 cases correspond to hospital admittance. Therefore, we use both confirm cases and deceased counts for our inferences, as explained in the previous section. Regarding data availability for our observational model, we use the patient’s reported onset of symptoms date. Due to administrative reporting delays, we discard the last 11 days of reporting and add four days as the time stamp for hospital admittance. We call this procedure the -11+4 data correction for reporting delays. We use the registered deceased date as the timestamp for death counts.

We consider daily deaths counts *d*_*i*_ and its theoretical expectation that is estimated in terms of the dynamical model as *μ*_*D*_(*t*_*i*_) = *D*(*t*_*i*_) − *D*(*t*_*i*−1_) for the metropolitan area or region being analyzed. Analogously, we consider daily cases *c*_*i*_ and its corresponding *μ*_*c*_(*t*_*i*_) given by the daily flux entering the *H*^1^ compartment, which may be calculated as in [17], namely

$$\begin{array}{c} \mu_{c}\left(t_{i}\right)=\int_{t_{i-1}}^{t_{i}}g\sigma_{2}I_{m}^{S}\left(t\right)dt, \end{array}$$

where $I_{m}^{S}\left(t\right)$ is the last state variable in the *I*^*S*^ Erlang series. We calculate the above integral using a simple trapezoidal rule with 10 points (1/10 day).

### Bayesian inference

To carry out a likelihood-based analysis, we assume that epidemic data has more variation than implied by a standard Poisson process, as is the case in other ecological studies. Following [16], we postulate that the number of both the confirmed cases and deaths follows a negative binomial distribution *NB*. Denoting the mean and variance as *μ* and *σ*^2^, and requiring that *σ*^2^ = *θ*_1_ *μ* + *θ*_2_ *μ*^2^ > *μ* we enforce overdispersion for suitable chosen parameters *θ*_1_ and *θ*_2_. For data *y*_*i*_, namely *c*_*i*_ and *d*_*i*_, we reparametrize the negative binomial distribution and let *y*_*i*_ ∼ *NB*(*pμ*(*t*_*i*_), *θ*_1_, *θ*_2_), with fixed values for the overdispersion parameters *θ*_1_, *θ*_2_ and an additional reporting probability *p*. The reporting probability for incidence was set to 0.85 and for deaths to 0.95. In general, it is more probable that people fail to seek treatment in a hospital (and thus tested and counted as a confirmed case) than missing to count a COVID19 related hospital death; see the S1 File for further details.

We assume conditional independence in the data, and therefore from the NB model, we obtain a likelihood. Our parameters are the contact rate parameter *β*’s, the *ω*’s and crucially we also infer the initial conditions *E*(0), *I*^*A*^(0), *I*^*S*^(0). Letting *S*(0) = *N* − (*E*(0) + *I*^*A*^(0) + *I*^*S*^(0)) and setting the rest of the parameters to zero, we have all initial conditions defined and the model can be solved numerically to obtain *μ*_*D*_ and *μ*_*c*_ to evaluate our likelihood.

Finally, regarding the elicitation of the parameters prior distribution, we use *Gamma* distributions with scale 1 and shape parameter 10 to model the initial conditions *E*(0), *I*^*A*^(0), *I*^*S*^(0) of the community transmission. Following the argumentation of Cori *et al*. [40], we assume that local transmission starts when there are 10 confirmed cases. The rationale for using Gamma distribution priors is that we can specify the distribution by prescribing its first two moments, and the resulting distribution verifies a maximum entropy condition. Namely, we obtain the less informative distribution that has the prescribed mean and (log) variance [43]. The prior for the first transmission rate *β*_0_, is a long tail, log Normal with *σ*^2^ = 1 and scale parameter 1; that is *log*(*β*_0_) ∼ *N*(0, 1). For the subsequent *β*’s, we use autoregressive priors to impose some coherence from one change point to the next with *log*(*β*_*i*_) ∼ *N*(*log*(*β*_*i*−1_), 1). The prior on *ω*_*i*_ is a *Beta*(1 + 1/6, 1 + 1/3) restricted to *ω*_*i*_ > *ω*_*i*−1_. This beta distribution is a fairly flat near uniform density in [0, 1], touches zero in 0 and 1, and is slightly skewed to lower values. We model here the unlikely values *ω*_*i*_ = 0, 1 and that under current social distancing measures we expect smaller rather than larger *N*_*eff*_. Otherwise, the prior for the *ω*_*i*_’s is rather diffuse and non-informative (see also S5 Table in S1 File).

To sample from the posterior we resort to MCMC using the “t-walk” generic sampler [44]. The MCMC runs semi-automatic, with consistent performances in most data sets.

### Displaying results

As in the case of climate forecasting, due to the stochastic nature of a pandemic outbreak point-wise estimates such as the maximum a posteriori estimate (MAP) does not provide good descriptions of the outbreak evolution. No single trajectory of the SEIRD model provides a good description of the outbreak evolution, nor give accurate forecasts. Instead, to illustrate posterior uncertainty we resort to sequentially plotting some inter quantile ranges, as we explain next.

**Fig 2:**
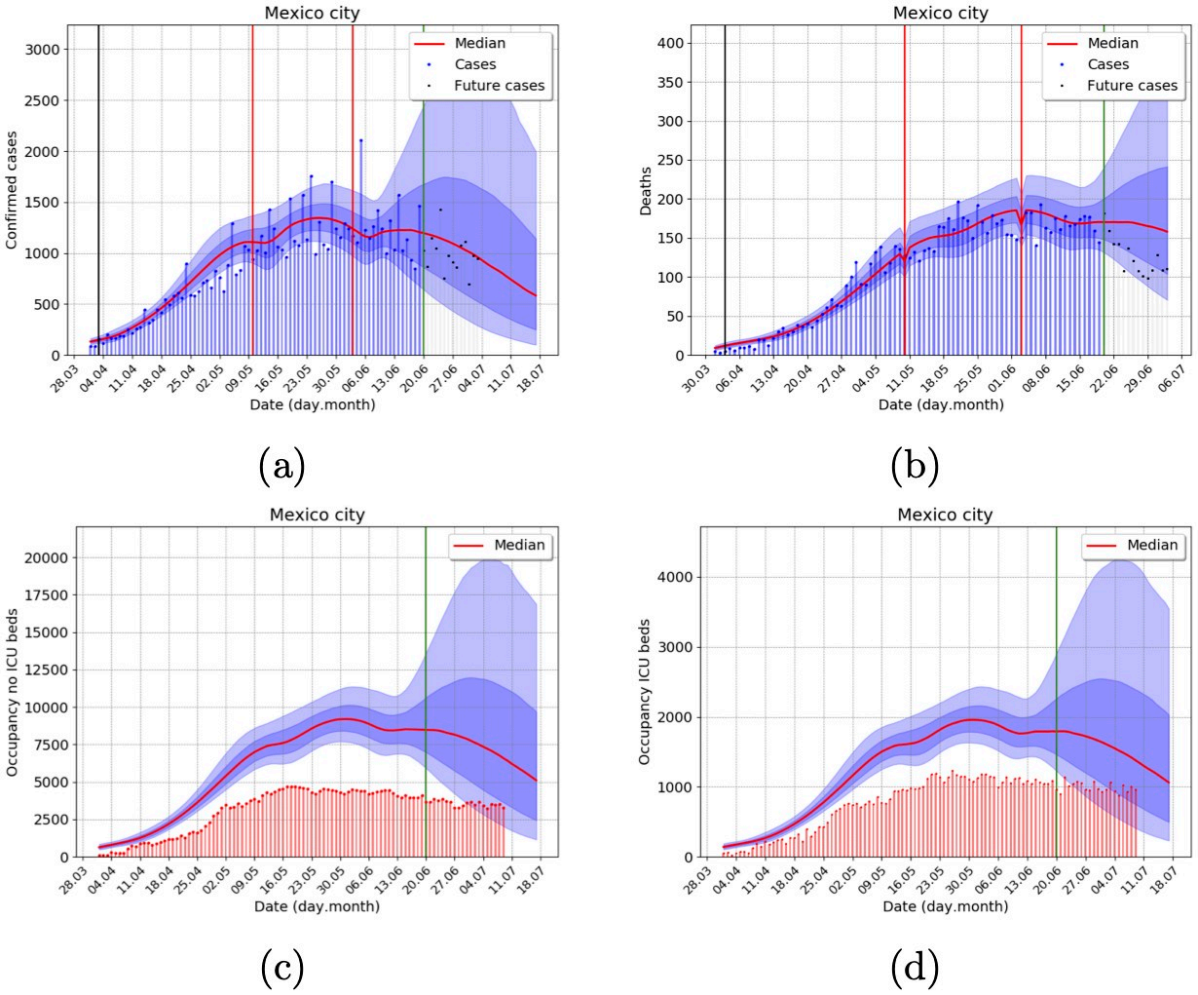
Retrospective outbreak analysis for Mexico city metropolitan area, considering data until 9 July 2020, with the -11+4 data correction for reporting delays explained in the observational model and data section. Posterior uncertainty is illustrated with the blue shadow areas, as explained in the Displaying Results section. Gray bars and dots correspond to two weeks of trimmed data, and the inference was done with blue data (blue bars and dots) only. The green vertical line shows the corresponding start date of forecasts. (A) Incidence of confirmed cases, (B) Incidence of deaths (C) No ICU, and (D) ICU demand of hospital beds. Actual hospital occupancy (red) is not used in the inference and does run until 9 July 2020. Total population 21, 942, 666 inhabitants.

**Fig 3:**
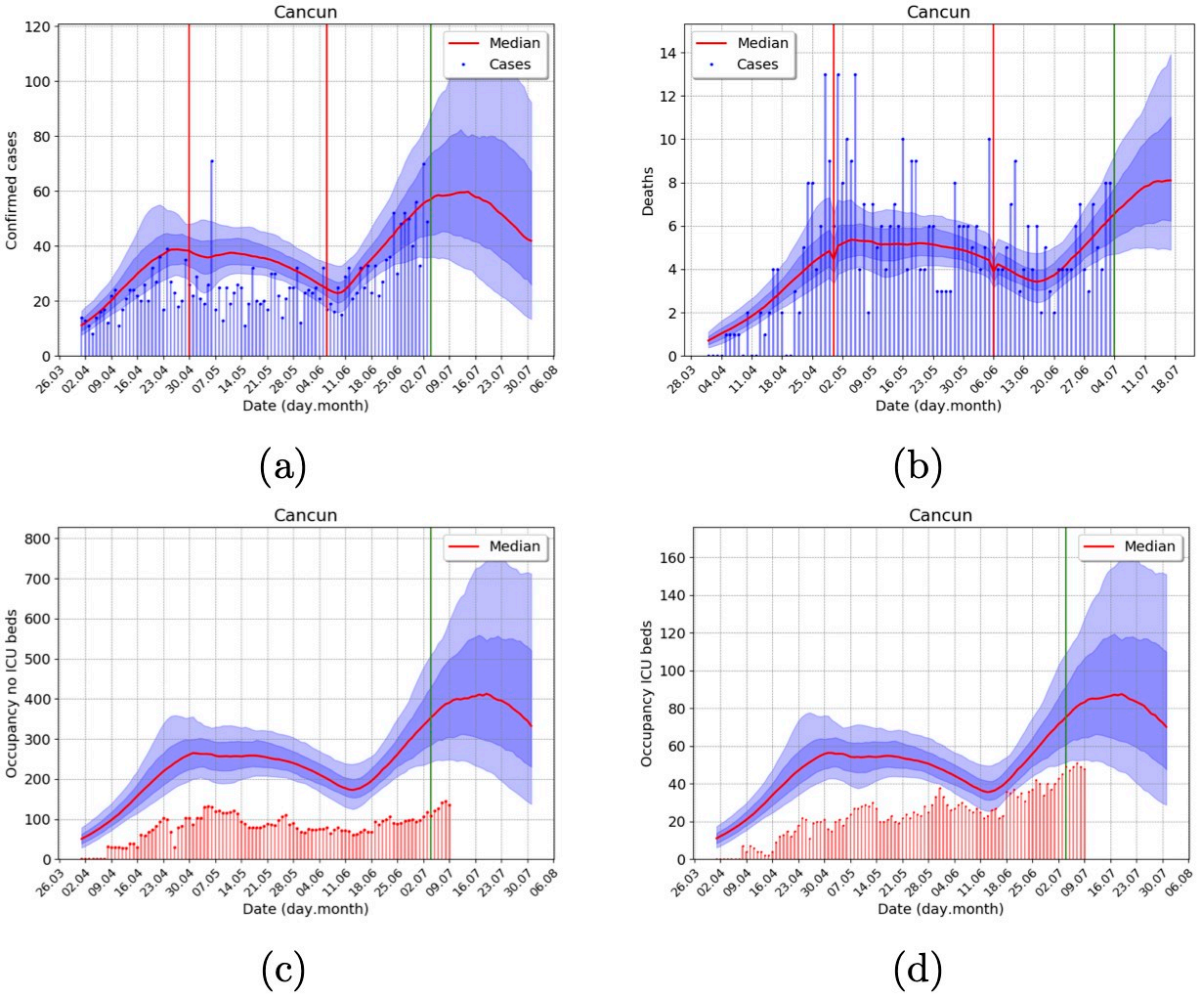
Outbreak analysis for Cancun metropolitan area, using data from 9 July 2020, with the -11+4 data correction for reporting delays. Posterior uncertainty is illustrated with the blue shadow areas, as explained in the Displaying Results section. The green vertical line shows the corresponding start date of forecasts. (A) Incidence of confirmed cases, (B) Incidence of deaths (C) No ICU, and (D) ICU demand of hospital beds. Total population 891, 843 inhabitants.

For any state variable *V*, the MCMC allows us to sample from the posterior predictive distribution for *V*(*t*_*i*_). By plotting some of its quantiles sequentially, we may produce predictions with quantified probabilistic uncertainty. In all model plots (Figs 2 and 3 and S1-S5 Figs in S1 File), the posterior predictive distribution at each day is plotted using the 10%—90% (light blue shadow) and 25%—75% (dark blue) quantile ranges. The corresponding median is the red trajectory. For example, there is a 50% posterior probability that the model trajectory lays within the dark blue area. Other basic posterior probabilities may be easily calculated.

## Results

Local transmission started at different dates in each Mexican metropolitan area, given the different communicability with Mexico City and the rest of the world. On the other hand, a country-wide general lockdown started on 22 May until 1 June, where each state started differently local control measures. All analyses presented here were done with data until 9 July 2020, with the -11+4 reporting delays strategy explained above. The total hospital bed occupancy estimate corresponds to the daily integral of *H*^1^ and *H*^2^ in the model, and the ICU occupancy corresponds to the *U*^1^ daily integral.

We calibrate residence times from reports on daily demand of hospital beds and intensive care unit records from *Instituto Mexicano del Seguro Social* or Mexican Social Security Institute (IMSS) at the early stages of the outbreak. In every case, we proposed each parameter value, namely hospital residence times, to overestimate the forecasted hospital bed and ICU demand intentionally. Accordingly, our results show a consistent overestimation and a shift to earlier times.

Fig 2A shows the model forecast, with quantified uncertainty, of the daily records of COVID-19 confirmed cases in Mexico City. Gray bars correspond to two weeks of trimmed data to assess the model performance. Fig 2B depicts records and forecasts incidence of deaths. In Fig 2C and 2D we compare the model forecasts with hospital bed and ICU occupancy obtained from a secondary official source of epidemiological surveillance depicted as red bars. Notice that the forecast begins after June 20th, and an uncertainty cone opens to the right the next 3 to 4 weeks. However, the attractor of the dynamical system closes the cone for longer times, and the predictive power of our forecast decreases. In Fig 2A and 2B, black and red vertical lines represent lockdown and relaxation days, respectively. Our model forecasts three different bump-shape regions where the effective population size increases.

We also present the case of Cancun’s metropolitan area since it is a medium-sized city with considerable international connectivity that was among the first ones with an outbreak in Mexico, see Fig 3. The forecast shows a clear first wave, with a long decreasing tail and a lockdown-induced 2nd wave after a lockdown easing and reopening of touristic activities.

**Fig 4:**
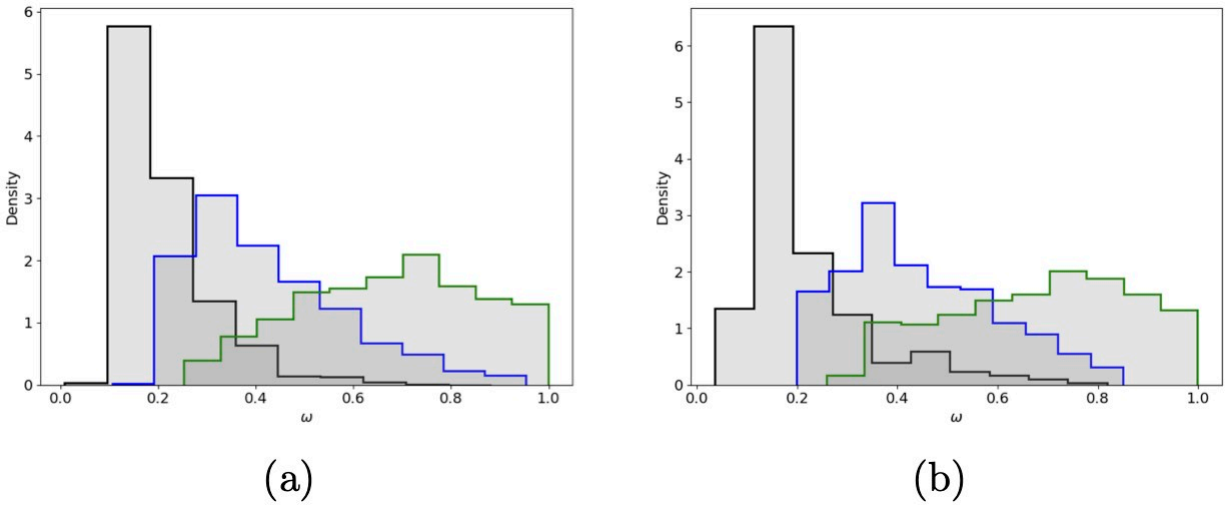
Posterior distribution for *ω*_0_ (black), *ω*_1_ (blue) and *ω*_2_ (green, *N*_*eff*_ = *Nω*_*i*_, *f* = 0.4) (A) Mexico city (B) Cancun.

**Fig 5:**
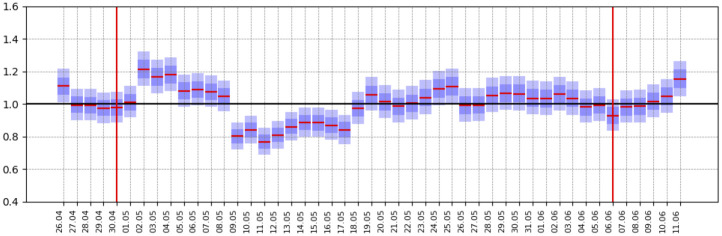
Daily *R*_*t*_’s calculated as in [40] for Cancun metropolitan area. Two relaxation days, marked with red vertical lines, were included at local minima, allowing for a minimum gap of 3 weeks. Blue shadow areas also represent the 10%—90% and 25%—75% quantile ranges, here for the daily *R*_*t*_ posterior distribution calculated as in [40].

In Fig 4 we show the three posterior distributions for *ω*. In (A), we show the Mexico City case, and in (B), we show the Cancun metropolitan area case. In Fig 5 we show the estimated Rt series as in [40] and the selected relaxation days for Cancun metropolitan area. In the S1 File, we show the outbreak analysis for some other cities to illustrate different aspects of our forecasting model’s performance. Besides this paper’s examples, we apply our model to 70 metropolitan areas and the 32 states in Mexico (“ama” model; https://coronavirus.conacyt.mx/proyectos/ama.html, in Spanish).

## Discussion

We present a SEIRD model to make probabilistic hospital pressure forecasts during COVID-19 outbreaks in metropolitan areas. We also consider lockdowns and lockdown-relaxations as two different kinds of interventions. We model the former as a change in transmission rates while the latter also allows for changes in the effective population size. These changes in effective population size and transmission rates are used as a proxy of behavioral changes, changes in the connectivity between population clusters, and super spreading events.

Our observation model is designed to integrate data after the nonlinear term in the flow diagram of the dynamic model (see Fig 1), and the rest of the dynamics is proportional to the hospital occupancy curves; therefore, the model forecasts can be used as a proxy of the full outbreak.

The underlying assumption that social contact and other conditions remain constant is not reasonable for most societies over long periods (e.g., more than eight weeks). Conservative short to mid-term forecasts must be preferred and change points added when necessary. To inform our model about intervention and relaxation days, we monitor in-line exogenous changes in SEIRD parameters. We offer both a theoretical and a numerical argument, see S8 Fig in S1 File, to show that setting relaxation days at local minima of *R*_*t*_ gives rise to robust inferences. Our approach yields a smaller dimension parameter space and a more practical forecasting algorithm.

Our choice of model’s parameter values was influenced by the evidence of the intense hospital pressure in places like Spain or Italy early in the pandemic. In every case, we proposed each parameter value to overestimate the forecasted hospital pressure. We could have chosen a different set of parameters resulting in a more accurate forecast but decided not to do so. Hence, the model over-estimates the hospital pressure. Despite this, the model’s sensible upper bounds are useful for public health. Underestimates of hospital demands would lead to poor planning, with possible hospital saturation and ICU bed shortage. Note that, as far as decision-makers are concerned, sensible upper bounds are preferred over underestimates of hospital demands. Underestimates of hospital demands would lead to poor planning, with possible hospital saturation and ICU bed shortage. Nevertheless, in the case of an overwhelmed hospital system, hospital residence times and Erlang series should be modified. However, up to our knowledge and according to Mexican health authorities, the highest reported ICU and hospital bed occupancy in all Mexican States was about 80% and 95%, respectively. There were some hospitals at full capacity, but fortunately, no official or news reports of any metropolitan area or state with its health care system overwhelmed. Hence we did not need such an analysis. However, Erlang densities may not correctly approximate residence times in some cases, and more general distributions should be considered. Moreover, as health professionals learn to treat the disease, hospital residence times also change. Both these effects should also be considered to obtain more accurate estimates in the outbreak’s long-term picture.

**Fig 6:**
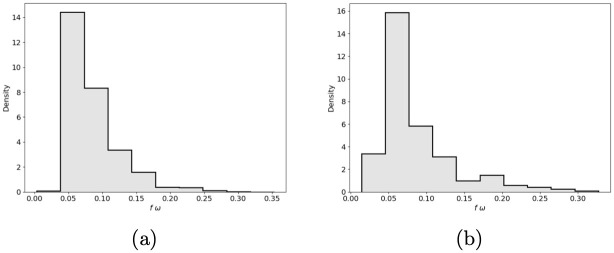
Posterior distribution of *f* × *ω* for (A) Mexico city and (B) Cancun metropolitan areas. With *f* = 0.4, the maximum a posterior of *f* × *ω* is close to 0.05 as proposed for the early forecast.

Interaction between the confounding effect and the dynamical model’s saturation mechanism has implications on the inference before and after the system arrives at its first peak. Although we can infer the contagious rate *β* at the early stages of the first epidemic wave, it is impossible to estimate effective population size (or even the product *ω* times *f*) from the data of confirmed cases and deaths. Not until saturation’s effects due to system size are noticeable, inferences on *ω* become viable (given *f*). Since forecasting is a time-continuous process, the above observations imply that we need to adapt our modeling strategy before and after the first peak. Before, we have to postulate the product *ω* × *f* or their separated values, and after we only need to postulate *f*. Notice that at the beginning of the epidemic outbreak in Mexico, since *f* is a disease-related parameter and *ω* is a proxy of the population’s response to health authority measures, to postulate their values is a delicate process. By modeling several countries that already had passed their first peak in April, we set *ω* = 1 and *f* = 0.05. This particular choice of parameters yielded some controversy that even reached the news media [45], but proved its validity afterwards. Once most Mexican cities pass the peak, we use our methodology by setting *f* to infer *ω* and compute the posterior distribution of the product *ω* × *f*. For *f* = 0.4, the maximum inferred posterior values of *ω* × *f* lay between 0.04 and 0.12 as can be seen in Fig 6. Notice that, due to the confounding effect, the inferred value of *ω* × *f* is independent of *f*’s value. The above difference between after and before the peak is important; forecasting models that fail to recognize this will consequently fail on their peak estimates.

We offer the forecasting performance in the *Results* section and **SM** ample evidence that our approach provides a reliable forecast at least two weeks ahead of the forecasting date. Our analysis showed that we account for the outbreak evolution in many metropolitan areas by setting one lockdown on 22 March of 2020, the beginning of country-wide lockdown, and two more relaxation days. One around 10 May of 2020, mother’s day, a popular celebration in Mexican culture where families gather together. The other relaxation day is set around four to eight days after 1 June of 2020, the announced date for the country-wide lockdown relaxation measures. In some cases, we also impose somewhat different relaxation days to account for local changes, such as the opening of tourist activity.

We measure the model’s forecasting performance in terms of how accurate and timely the predictions were compared with the actual phenomena *a posteriori*. In forty-five days forecast with an observation window of forty-five days, our model produced sensible probabilistic upper bounds on hospital-bed and ICU units demand in 87.5% and 75% of the cases, respectively (see S2 in the S1 File for the analysis of the largest 32 Mexican cities). Moreover, all predictions were also timely delivered before the maximum occupancy event in all cases. In this sense, we claim that our forecasts have been accurate and, more importantly, useful.

The confounding effect between the population size, namely *ω*, and the fraction of asymptomatic/mild–symptomatic infections 1 − *f* makes it impossible to forecast the population that will be in contact with the virus at the end of an outbreak reliably. Likewise, although it is possible to make a model-based analysis of scenarios of lockdown exit strategies, scenario estimation is limited due to the lack of information regarding population viral seroprevalence. Therefore, without serological studies in the open population after a COVID-19 outbreak, it is impossible to assess the final outbreak size.

Up to our knowledge, there are very few models that can produce forecast lockdown-induced 2nd waves with quantified uncertainty. Although more elaborate models can be considered, our model is simple and flexible enough to deliver reliable and useful forecasts.

## Supporting information

S1 File (PDF)
