# Supplementary material for: Forecasting hospital demand in metropolitan areas during the current COVID-19 pandemic and estimates of lockdown-induced 2nd waves
Source: PLoS One. 2021 Jan 22;16(1):e0245669. doi: 10.1371/journal.pone.0245669 (PMC7822260; doi:10.1371/journal.pone.0245669)
Supplement: S1 File — (PDF) [file pone.0245669.s001.pdf]

Forecasting hospital demand in metropolitan areas during the current COVID-19 pandemic and estimates of lockdown-induced 2nd waves

Marcos A Capistran[1*◐], Antonio Capella[2◐], and J. Andrés Christen[1◐].

1 Centro de Investigación en Matemáticas, CIMAT-CONACYT, Guanajuato, Guanajuato, Mexico.
2 Instituto de Matemáticas, UNAM, Circuito Exterior, CU, CDMX, Mexico.

◐These authors contributed equally to this work.
* marcos@cimat.mx

List of Supplementary Materials

S1 Other examples: Toluca, Merida, Cuernavaca, Acapulco, Culiacan
Figures S1-S5
S2 Mode forecasting performance
S3 Stability in choosing change points
S4 Model
S5 Data and observational model
S6 Modeling interventions and Bayesian inference
S7 Prior elicitation
S8 Confounding effect of $N_{eff} \times f$
S9 Data sources

S1 Other examples

S1.1 Toluca

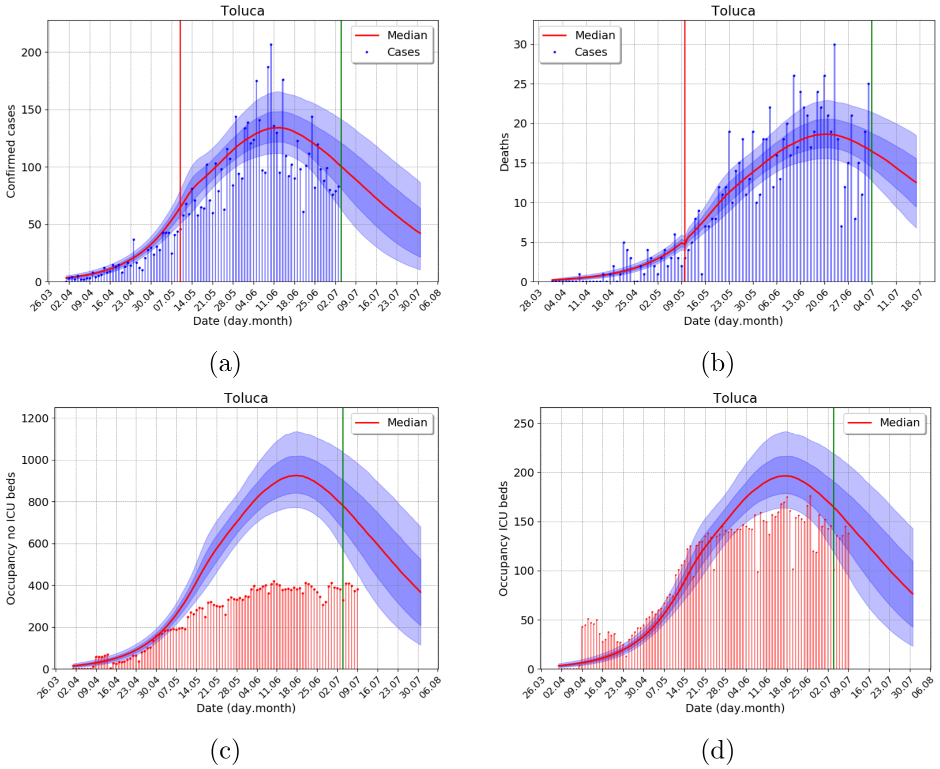

Fig S1. Outbreak analysis for Toluca (the state of Mexico, Mexico central highlands) metropolitan area, using data from 9 July 2020, with the -11+4 data correction for reporting delays explained in the main text. Posterior uncertainty is illustrated with the blue shadow areas, as explained in the Displaying Results section. The green vertical line shows the corresponding start date of forecasts. (a) Incidence of confirmed cases, (b) Incidence of deaths (c) No ICU, and (d) ICU demand of hospital beds. Total population $2,377,828$ inhabitants. Forecast for the city of Toluca is in course with one relaxation event.

S1.2 Merida

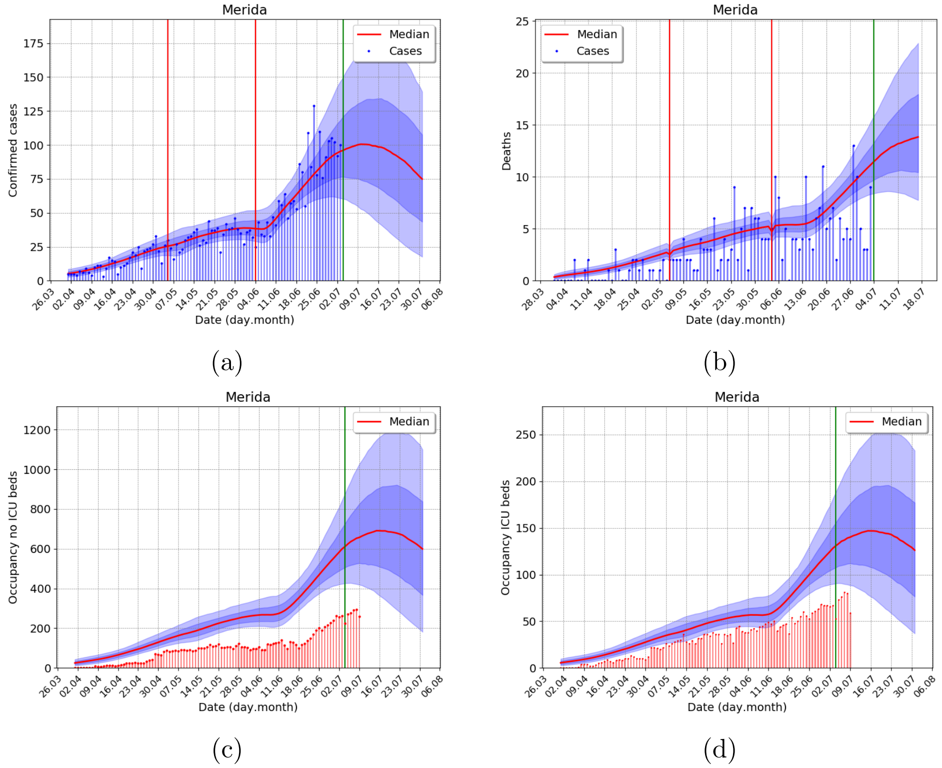

Fig S2. Outbreak analysis for Merida (state of Yucatan, Yucatan peninsula) metropolitan area, using data from 9 July 2020, with the -11+4 data correction for reporting delays explained in the main text. Posterior uncertainty is illustrated with the blue shadow areas, as explained in the Displaying Results section. The green vertical line shows the corresponding start date of forecasts . (a) Incidence of confirmed cases, (b) Incidence of deaths (c) No ICU, and (d) ICU demand of hospital beds. Total population $1,237,697$ inhabitants. There is an evident ongoing second outbreak in the city of Merida.

S1.3 Cuernavaca

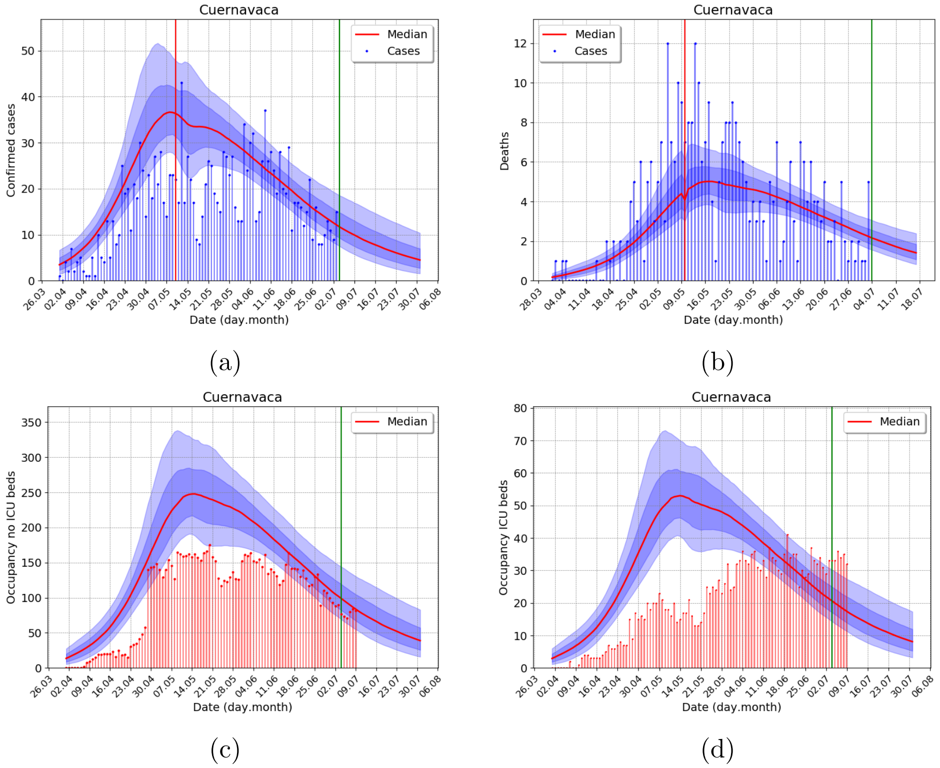

Fig S3. Outbreak analysis for Cuernavaca (the state of Morelos, Mexico central highlands) metropolitan area, using data from 9 July 2020, with the -11+4 data correction for reporting delays explained in the main text. Posterior uncertainty is illustrated with the blue shadow areas, as explained in the Displaying Results section. The green vertical line shows the corresponding start date of forecasts. (a) Incidence of confirmed cases, (b) Incidence of deaths (c) No ICU, and (d) ICU demand of hospital beds. Total population $1,059,521$ inhabitants. In the city of Cuernavaca, the model captures the slow decline of the outbreak.

S1.4 Acapulco

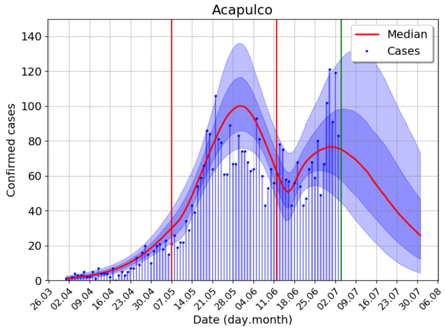

(a)

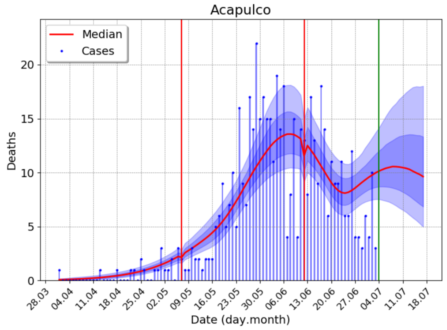

(b)

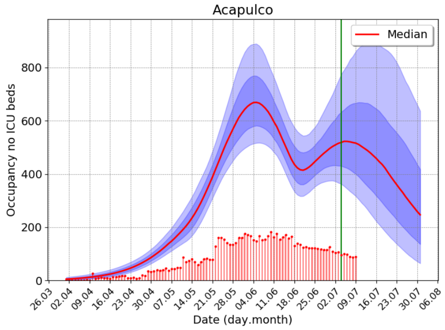

(c)

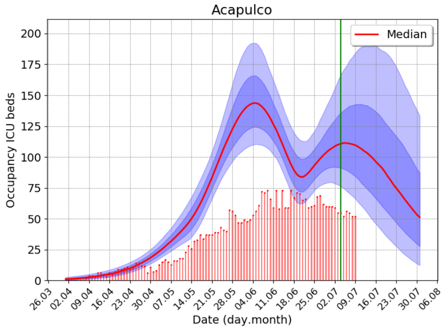

(d)

Fig S4. Outbreak analysis for Acapulco (the state of Guerrero, Mexico south pacific shore) metropolitan area, using data from 9 July 2020, with the -11+4 data correction for reporting delays explained in the main text. Posterior uncertainty is illustrated with the blue shadow areas, as explained in the Displaying Results section. The green vertical line shows the corresponding start date of forecasts. (a) Incidence of confirmed cases, (b) Incidence of deaths, (c) No ICU, and (d) ICU demand of hospital beds. Total population $1,059,521$ inhabitants. The outbreak in the city of Acapulco is an example of a second outbreak of the same size.

S1.5 Culiacan

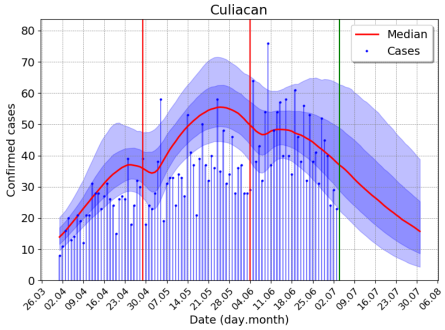

(a)

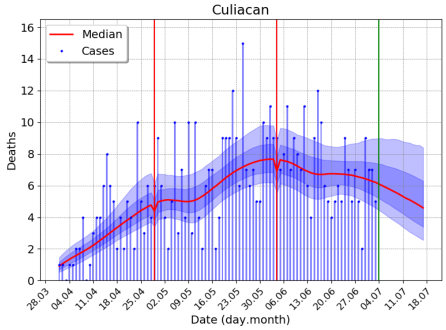

(b)

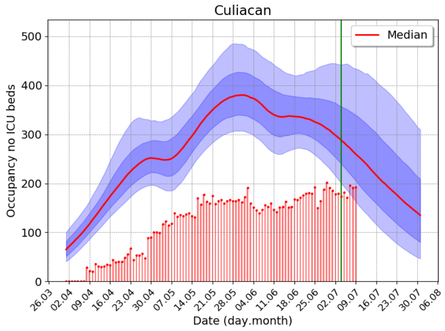

(c)

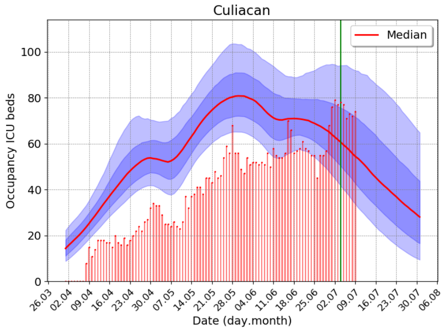

(d)

Fig S5. Outbreak analysis for Culiacan (the state of Sinaloa, Mexican north pacific shore) metropolitan area, using data from 9 July 2020, with the -11+4 data correction for reporting delays explained in the main text. Posterior uncertainty is illustrated with the blue shadow areas, as explained in the Displaying Results section. The green vertical line shows the corresponding start date of forecasts. (a) Incidence of confirmed cases, (b) Incidence of deaths, (c) No ICU, and (d) ICU demand of hospital beds. Total population 962, 871 inhabitants. Example of a secondary outbreak with two lockdown-induced second waves.

S2 Model forecasting performance

The QoI in the forecast is the number of hospital and ICU/respiratory support beds needed in a metropolitan area. We measure the model's forecast performance by comparing observed occupancy with the model's probabilistic upper bounds in an observation window. In figures S6 and S7, we show this comparison for sixty days forecast of maximum occupancy with the observed occupancy forty-five days after the forecast. Cities are sorted by size, and the vertical lines separate them into three groups; smaller than 1 million, between 1 million and 2.5 million, and larger than 5 million inhabitants. The last column in both graphs corresponds to Mexico City, a 22 million inhabitant metropolitan area.

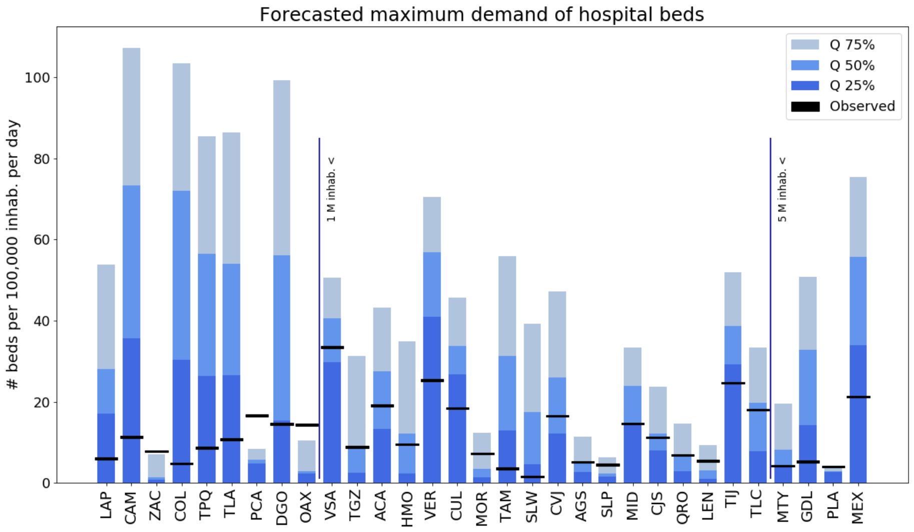

Fig S6. Forty-five forecast of maximum hospital beds demand in the country's largest 32 cities with data up to 15 April 2020. Horizontal black lines represent the maximum observed hospital bed occupancy during the next 45 days after the forecast.

The interpretation of figures S6 and S7 is as follows: for each bar, the blue-tones represent an upper bound on the maximum number of beds that will be occupied with a probability of Q percent. That is, the figures shows the estimated upper bounds of hospital demand in the forecasting period as probability distributions. For instance, the graph in figure S6 shows that in Mexico city's metropolitan area (MEX), there is was a 25% probability that the actual hospital bed demand is above 74 beds per 100,000 inhabitants per day in the 45 days of the forecasting window. Health authorities used these estimates in the emergency planning. In this case, satisfactory performance means that the observed bed demand was below our estimated upper bounds in the forecasting window.

For hospital beds, forecast occupancy falls under the 50% quantile 24 times and under the 75% quantile on four occasions. In the ICU case, forecast occupancy falls under 50% and 75% quantiles 13 and 9 times, respectively. The model's forecast underestimated ICU and hospital bed demand, namely above 75% quantile in eight and three cases, respectively.

For cities under 1 million inhabitants, the uncertainty's forecast is systematically bigger than for larger cities. In absolute terms, i.e., the number of beds needed in a metropolitan area, more uncertainty in smaller metropolitan areas is less critical in the

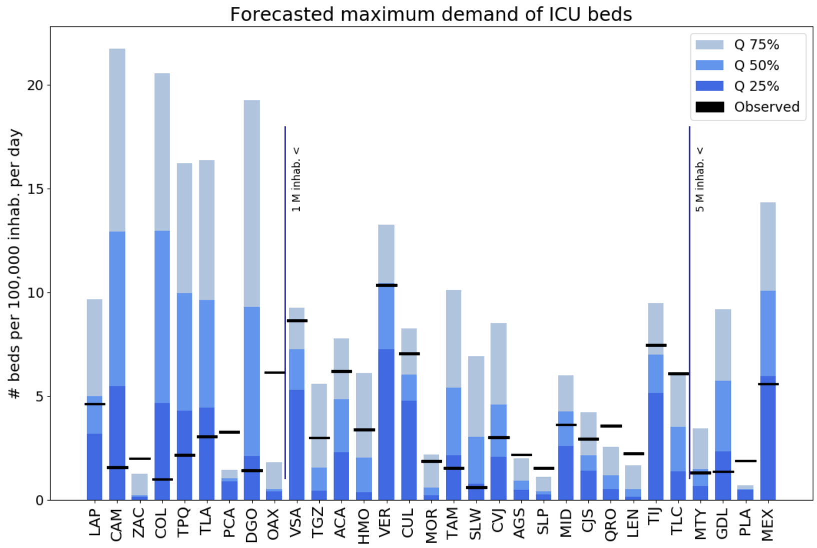

Fig S7. Forty-five forecast of ICU beds demands in the country's largest 32 cities with data up to 15 April 2020. Horizontal black lines represent the maximum observed hospital bed occupancy during the next 45 days after the forecast.

emergency response plans. A forecasted occupancy of 10 ICU beds per 100,000 inhabitants in a city of 0.5 million is about 50 ICU beds, while in Mexico City metropolitan area, the same forecast yields about 2,200 ICU beds.

Key of accronims in figures S6 and S7: LAP: La Paz, CAM: Campeche, Zac: Zacatecas, COL: Colima, TPQ: Tepic, TLA: Tlaxcala, PCA: Pachuca, DGO: Durango, OAX: Oaxaca, VSA: Villahermosa, TGZ: Tuxtla Gutierrez, ACA: Acapulco, HMO: Hermosillo, VER: Veracruz, CUL: Culican, MOR: Morelia, TAM: Tampico, SLW: Saltillo, CVJ: Cuernavaca, AGS: Aguscalientes, SLP: San Luis Potosi, MID: Merida, CJS: Ciudad Juarez, QRO: Queretaro, LEN: Leon, TIJ: Tijuana, TLC: Toluca, MTY: Monterrey, GDL: Guadalajara, PLA: Puebla-Tlaxcala, and MEX: Mexico City metropolitan area.

S3 Stability in choosing change points

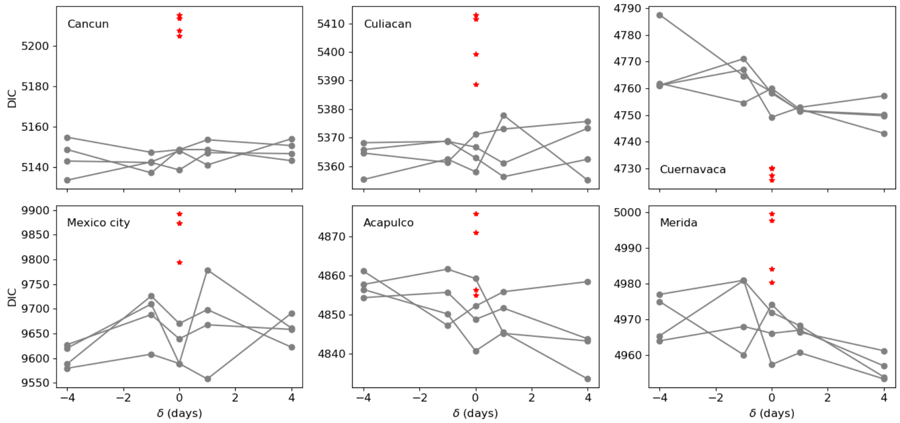

Fig S8. Deviance Information Criterion (DIC, smaller values are preferred) calculated for a shift of δ days of the last relaxation day used in the corresponding examples, and without the relaxation day (red asterisk). We repeated the calculations four times with 10^6 MCMC iterations each to capture the MC error. Toluca (not shown) exhibits a similar DIC profile as Cancun.

As explained in the main text, we include relaxation days based on local minima of the R_t's (see Fig. 5). Relaxation points indicate that a larger ω needs to be included, signifying, in turn, that more population participates in the epidemic. Precisely, a local minimum in the R_t suggests a change in the contagion pattern and a new infection wave.

A more formal approach could be attempted to include relaxation points. The number of relaxations days may be a parameter itself, trying to infer the number of change points and their positions. This would result in a transdimensional MCMC [1] with several orders of magnitude in complexity. The inference process is already complex, and as more data arrives, it becomes more CPU intensive. Moreover, transdimensional MCMC's, as the Reversible Jump MCMC [1], are challenging to set up and tune and more or less automatic versions as the t-walk are not as yet available. Considering the ongoing production of 70+ weekly reports, running the transdimensional MCMC is unfeasible.

As explained in the main text, we took a more practical approach considering it a modeling problem, using an independent score as the R_t's to nominate change points. While the procedure seems pragmatically reasonable, the actual minima of the R_t's are not clear since they are also inferred [2]. Some judgment is required to choose among neighboring days. This raises a concern of the robustness in choosing possible relaxation days, questioning results if the actual choice is shifted some days before or after.

A compromise between the full-blown transdimensional MCMC and our judiciously choosing the R_t would be to use an information type criterion to choose among a vicinity of relaxation days around a R_t minimum vs not including a change point. This will shed light on the robustness of our approach.

Some model selection criteria have been proposed, for example, the Akaike Information Criterion (AIC) or its Bayesian counterpart, the Bayesian Information Criterion (BIC). For technical reasons, the Deviance Information Criterion (DIC) has been proposed as more adequate for Bayesian applications [3]. The crucial difference between the BIC (or the AIC) and the DIC is that the former is calculated pointwise at the log posterior (log-likelihood) maximum, whereas the latter comprises a posterior

expected value of the log-likelihood; see [3] for details. The DIC does provide more reliable results for model comparisons, but the involved expected value, although straightforward to approximate using the MCMC sample, maybe an estimation problem itself.

We performed an experiment using the DIC in the seven metropolitan areas presented in the paper; see Fig. S8. The DIC for the relaxation day shifted four and one days before and after, and the model without the relaxation day, is always quite close. In fact, the DIC's are so close that we required a substantial MCMC sample to estimate the DIC to sufficiently indicate a higher or lower DIC for the included model choices. We needed to run the MCMC to 10^6 iterations for minimum stable results, and we ran these four times to appreciate the Monte Carlo error involved, see Fig. S8. It took approximately 12 hours running in 6 processors to complete the computation for each Metropolitan area. Fig. S8 took us several days to complete.

The DIC analysis seems computationally infeasible as well, but the experiment here performed shows that the process of choosing a relaxation day is not too sensitive to the exact chosen day. In Fig. S8 we see that 1) the DIC of models resulting from shifted relaxation days are always quite close, relative to the DIC of the model without the change point (red asterisk). Our computations suggest that the choice is stable and robust and may vary within a reasonable range, obtaining similar results. 2) The DIC for the model without the relaxation day is above or close to the DIC's including the change point, the only exception being Cuernavaca.

Although pragmatic and with user input required, our approach to choosing relaxation days seems robust and provides a workable alternative to otherwise prohibitive alternatives.

Table S1. Description of the state variables in the dynamic model

Variable	Description
S	Susceptibles
E	Latent individuals
I^A	Asymptomatic/mild–symptomatic individuals
I^S	Symptomatic individuals
I^C	Out-patients
H^1	Hospitalized patients, initial stage
H^2	Hospitalized patients (no ICU)
U^1	Hospitalized patients (ICU or respiratory support)
U^2	Hospitalized patients (ICU or respiratory support) critical day
H^3	Hospitalized patients recovering after ICU or respiratory support
R	Recovered
D	Deceased

S4 Model

We developed a dynamic transmission compartmental model to simulate the spread of the novel coronavirus SARS-CoV-2. A definition of the state variables is given in Table S1. Additionally, an "Erlang series" is included for most of these state variables to account for non-exponential residence times. The model may be described conceptually with the graph in Fig 1. Without showing the Erlang series for the sub-compartments the system of equations in the model is as follows:

$$
\begin{aligned}
\frac{dS}{dt} &= -\frac{\left(\beta_A I^A + \beta_S I^S\right)}{\omega N} S & \frac{dE}{dt} &= \frac{\left(\beta_A I^A + \beta_S I^S\right)}{\omega N} S - \sigma_1 E \\
\frac{dI^A}{dt} &= (1-f)\sigma_1 E - \gamma_1 I^A & \frac{dI^S}{dt} &= f\sigma_1 E - \sigma_2 I^S \\
\frac{dI^C}{dt} &= (1-g)\sigma_2 I^S - \gamma_2 I^C & \frac{dH^1}{dt} &= g\sigma_2 I^S - \sigma_3 H^1 \\
\frac{dH^2}{dt} &= (1-h)\sigma_3 H^1 - \gamma_3 H^2 & \frac{dU^1}{dt} &= h\sigma_3 H^1 - \sigma_4 U^1 \\
\frac{dU^2}{dt} &= \sigma_4 U^1 - \mu U^2 & \frac{dD}{dt} &= i\mu U^2 \\
\frac{dH^3}{dt} &= (1-i)\mu U^2 - \gamma_4 H^3 \\
\frac{dR}{dt} &= \gamma_1 I^A + \gamma_2 I^C + \gamma_3 H^2 + \gamma_4 H^3 \quad .
\end{aligned}
$$

In Table S2, we give a brief description of all the parameters in the model.

S4.1 Infection force and basic reproductive number $\mathcal{R}_0$

For the infection force (λ) we assume that individuals that spread the infection correspond to the mild–symptomatic/asymptomatic (I^A) and symptomatic individuals (I^S) before they get contact with the health care system or doctor, e.g.

$$\lambda = \frac{\beta_A I^A + \beta_S I^S}{N_{eff}}$$

We compute the basic reproductive number R_0 of the epidemic by the next

Table S2. Description of model parameters.

Parameter	Description	Units
N	Total number individuals in the population	–
$\omega(t)$	Percentage susceptible individuals in the population at time t	–
$\beta_A(t)$	Transmission rate of asymptomatic/mild–symptomatic individuals (asx/mild–sym) at time t	per day
$\beta_S(t)$	Transmission rate of symptomatic individuals at time t	per day
κ	Relative strength between the transmission the rate of asx/mild–sym and symptomatic	–
f	Proportion of infected persons with strong enough symptoms to visit a hospital	–
g	Proportion of infected persons that need hospitalization	–
h	Proportion of hospitalized patients requiring respiratory support or ICU care	–
i	Proportion of Respiratory-assisted or ICU patients deceased	–
$1/\sigma_1$	Average incubation time	day
$1/\sigma_2$	Average time from symptomatic onset to hospital visit	day
$1/\sigma_3$	Average time from hospital admission to respiratory support or ICU care	day
$1/\sigma_4$	Average time with respiratory support or ICU care	day
$1/\mu$	Average length of critical stage of respiratory–support/ICU between death and recovery	day
$1/\gamma_1$	Average time that asymptomatic/mild–symptomatic individuals remain infectious	day
$1/\gamma_2$	Average time of symptomatic individuals that recover without visiting a hospital	day
$1/\gamma_3$	Average time from hospital admission to hospital discharge	day
$1/\gamma_4$	Average time from respiratory–support/ICU care release to hospital discharge	day

generation matrix method [4] and obtain

$$R_0 = (1-f)\frac{\beta_A}{\gamma_1} + f\frac{\beta_S}{\sigma_2}. \quad (1)$$

S4.2 Values of model parameters

Since our QoI are related to the hospital pressure we choose all parameters conservatively. For each metropolitan area, we assume that N corresponds to its full population, as defined by Instituto Nacional de Estadistica y Geografia (INEGI). The values of the transition probabilities are summarized in Table S3.

Table S3. Transition probabilities at bifurcations in the model

Parameter	Value	Reference
f	0.40	postulated
g	0.04375	[5], IMSS
h	0.25	[5], IMSS
i	0.5	[5], IMSS

S4.3 Erlang series and sub-compartments

To make the intrinsic generation-interval of the renewal equation in each compartment more realistic we divide each compartment of the model into m equal sub-compartments to generate an Erlang–distributed waiting time [6]. The Erlang distributions of each compartment is calibrated by two parameters: the rate λ_E and the shape m, a positive integer that corresponds to the number of sub-compartments on the model. In terms of these parameters the mean of the Erlang distribution is m/λ_E, this mean correspond to the average times in the dynamic model.

We use recent publications and information generously shared by the Instituto Mexicano de Seguridad Social (IMSS) to estimate the average time and the shape parameter of the Erlang series in each compartment. In Table S4, we give details of Erlang series lengths, residence times and imputed values.

Table S4. Average times and Erlang shape parameters for sub-compartments

Variable	Rates	Average time	Erlang shape m	Reference
S	β_S	Inferred	1	–
E	$1/\sigma_1$	5 days	4	[7]
I^A	$1/\gamma_1$	7 days	3	[8]
I^S	$1/\sigma_2$	4 days	3	[9]
I^C	$1/\gamma_1$	7 days	3	[10]
H^1	$1/\sigma_3$	2 days	10	[11]
H^2	$1/\gamma_3$	10 days	3	[11]
U^1	$1/\sigma_4$	10 days	3	IMSS
U^2	$1/\mu$	1 day	1	[10]
H^3	$1/\gamma_4$	4 days	5	[11]
R	None	–	–	–
D	None	–	–	–

S4.4 Relative strength between the transmission the rate of asymptomatic/mild–symptomatic and symptomatic

In our methodology, we aim to infer the force of the infection λ. This parameter is defined in terms of contact rate of asymptomatic/mild–symptomatic individuals β_A and contact rate of symptomatic individuals β_S. Due to the functional dependence of λ in these parameters, there is a lack of identifiability between β_A and β_S that can not be resolved without further assumptions. We assume that the relative strength between the transmission rate of asymptomatic/mild–symptomatic and symptomatic is modeled as a fixed ratio κ. We model the value of κ directly as the ratio of the viral load of symptomatic and asymptomatic/mild–symptomatic patients [12, 13] and fixed it to $\kappa = 2$. Hence, the force of infection becomes $\lambda = \beta^S(I^S + \kappa I^A)/N_{eff}$.

S5 Data and observational model

For inference, we, therefore, consider daily confirmed cases c_i of patients arriving at H^1 and daily reported deaths d_i, for the metropolitan area or region being analyzed.

The first default model for count data is a Poisson distribution; however, epidemiological data tends to be overdispersed. Thus, an over disperse generalized Poisson distribution may be needed to correctly, and safely, model these types of data. Following [14] (see main paper) the NB distribution is re parametrized in terms of its mean μ and "overdispersion" parameters θ and ω_{NB}, with $r = \frac{\mu}{\omega_{NB}-1+\theta\mu}$ and $p_{NB} = \frac{1}{\omega+\theta\mu}$, the number of failures before stopping and the success probability, respectively, in the usual NB parametrization. For data y_i we let $y_i \sim NB(p\mu(t_i), \omega, \theta)$, with fixed values for the overdispersion parameters ω_{NB}, θ and an additional reporting probability p. The index of dispersion is $\sigma^2/\mu = \omega_{NB} + \theta\mu$. Over dispersion with respect to the Poisson distribution is achieved when $\omega_{NB} > 1$ and the index of dispersion increases with size if $\theta \neq 0$; both desirable characteristics in outbreak data, adding variability as counts increase. In both cases we found good performance fixing $\omega_{NB} = 2$. To model daily deaths, we fixed $\theta = 0.5$ and for daily cases $\theta = 1$ implying higher variability for the later. The reporting probabilities are 0.95 for deaths and 0.85 for cases, with the assumption that the c_i's are confirmed sufficiently severe cases arriving at hospitals. As explained in the main paper, the theoretical expectations estimated in terms of the dynamical model are given by $\mu_D(t_i)$ and $\mu_c(t_i)$ for dead and cases, respectively.

S6 Modeling interventions and Bayesian inference

We assume conditional independence in the data, and therefore from the NB model, we obtain a likelihood. Our parameters are the contact rate parameters β's, the ω's and crucially we also infer the initial conditions $E(0), I^A(0), I^S(0)$. Letting $S(0) = N - (E(0) + I^A(0) + I^S(0))$ and setting the rest of the parameters to zero, we have all initial conditions defined and the model may be solved numerically to obtain μ_D and μ_c to evaluate our likelihood. We use the *lsoda* solver available in the *scipy.integrate.odeint* Python function.

Moreover, as explained in the main paper, we also estimate ω_i with $N_{eff} = \omega_i N$, both before and after a change point ("relaxation day"; we show intervention days with black vertical lines and relaxation change points with red vertical lines in our plots, see next section). To make it a bit simpler, we also force the model to have a new β parameter after a relaxation day. However, the process of numerically solving the system of ODE's is slightly more complex. For a set of parameter values, including the

ω, to evaluate the likelihood, one needs to apply the solver from time $t = 0$ to the first relaxation day, considering $N_{eff} = \omega_1 N$. Then for $\omega_2 > \omega_1$ we use the last values of all state variables as initial values for a second solve now with $N_{eff} = \omega_2 N$, and so forth.

To sample from the posterior, we resort to MCMC using the "t-walk" generic sampler [15]. The MCMC runs semi-automatic, with a reasonably consistent burn-in of 1,000 iterations (initial sampling values from the prior). We use the Integrated Autocorrelation Time (IAT) to assess the efficacy of the MCMC sample. The IAT estimates the number of iterations needed to achieve independence [16], namely the *thinning* or subsampling needed to obtain a pseudo-independent sample. As is typical with the t-walk, the IAT divided by the number of parameters remains around 30 to 50. We performed subsampling using the IAT, obtaining pseudo-independent sample sizes of 1,000 to 1,500 with 400,000 iterations of the MCMC. This process takes roughly 60 min in a 2.2 GHz processor.

To illustrate the whole posterior distribution, for any state variable V (or $\mu_c(t_i)$), for each sampled initial conditions and β's the model is solved at time $t_1, t_2, \dots, t_k$, including possibly future dates, obtaining a sample of $V(t_i)$ values for each t_i. The median and other desired quantiles are plotted vertically for each date considered, obtaining the plots as in Fig 2. Note that the traced median or other plotted quantiles do not necessarily correspond to any given model trajectory. It provides a far richer Uncertainty Quantification approach than the classical parameter estimates plug-in approach. Indeed, the sampled values for $V(t_i)$ do correspond to Monte Carlo samples of the posterior predictive distribution for $V(t_i)$.

S7 Prior elicitation

In the current setting, we have substantial knowledge about the model parameters to pose prior distribution models. Indeed, regarding the contact rate β_i there are rigorous analysis about R_0 such as Park *et al.* [17], which in our model is given by Eq. (1), to model the mean and variance of β_i. On the other hand, to select the mean and variance of the prior model distribution of the initial conditions $E(0)$,$I^A(0)$, and $I^S(0)$ we use the heuristic rule that local transmission starts when the number of cases is roughly 10, see Cori *et al.* [2]. We summarize the prior elicitation reasoning in Table S5.

S8 Confounding effect of $N_{eff} \times f$

To explain the confounding effect of $N_{eff} \times f$ we have two observations. First, if we let $f = \tilde{f}/\alpha$ for some $\alpha \in (0, \tilde{f})$ then differential equations for the variables I^c, H^1, H^2, H^3, U^1, U^2 and D remain invariant and the equations for I^s becomes

$$\frac{dI^S}{dt} = \frac{\tilde{f}}{\alpha}\sigma_1 E - \sigma_2 I^S.$$

By letting $\tilde{E} = E/\alpha$ the equation for I^s is also invariant with the substitution of E by $\tilde{E}$. Now, the equation for $\tilde{E}$ is given by

$$\frac{d\tilde{E}}{dt} = \frac{\left(\beta_A I^A + \beta_S I^S\right)}{\alpha N_{eff}} S - \sigma_1 \tilde{E}.$$

By letting $\tilde{N}_{eff} = \alpha N_{eff}$ the latter equations becomes also invariant under the substitution of E by $\tilde{E}$. Therefore for the lower branch in the model (see Fig 1) the system of equations is invariant under the change of f and N_{eff} by $\tilde{f}$ and $\tilde{N}_{eff}$

Parameter	Prior distribution	Rationale
$E(0)$,$I^A(0)$,$I^S(0)$	For initial conditions $E(0)$,$I^A(0)$, and $I^S(0)$ we pose a Gamma prior distribution with shape $k = 1$ and scale $\theta = 10$ parameters	The Gamma distribution is a maximum entropy distribution characterized by its mean $k\theta$ and variance $k\theta^2$, see [18]. We set the mean at the value often used to model the beginning of local transmission, see Cori *et al.* [2]
β_i	For the contact rate β_i we pose a Log-normal distribution with parameters $\mu_i = 1$ and $\sigma_i = 1$ for $i = 1$, corresponding to the beginning of the lockdown and $\mu_i = 1$ and $\sigma_i = \beta_i - 1$ for for subsequent forecasting events	For the first forecast we inform the expected value of the contact rate β using reported values of R_0 of places under lockdown. Subsequent values of the contact rate have an autoregresive prior model.
ω_i	For the effective population parameter ω_i, defined by $N_{eff} = \omega_i N$ we use a Beta distribution with shape parameters $\alpha = 5/6$ and $\beta = 4/3$	The effective population parameter can be inferred after the peak.

Table S5. We pose prior models on the contact rate and initial condition parameters using expert knowledge about R_0 and number of infected individuals at the beginning of community transmission.

provided $\tilde{N}_{eff} \times \tilde{f} = N_{eff} \times f$ holds. We need to adapt the equations for S, I^A, and R to get a consistent system of equations.

Second, to infer parameter β we inform the system with data at H^1 and D compartments. If $\tilde{N}_{eff} \times \tilde{f} = N_{eff} \times f$ holds, in view of our first observation, to fit these data the fluxes $f\sigma_1 E$ and $\tilde{f}\sigma_1\tilde{E}$ in either case have to be the same. The solutions in the compartment I^S and after do not change in this case, but the individuals in the I^A compartment does change depending on which combination of N_{eff} and f or $\tilde{N}_{eff}$ and $\tilde{f}$ is considered. There is a range of validity for α where the inference of β does not change, but we do not explore this property further.

We also present numerical simulations to confirm this confounding effect (see Fig S9). While the asymptomatic infection is not fully described, this confounding issue will remain unsolved.

S9 Data sources

We take metropolitan areas delimitation and population from [19] and [20], respectively. Official records of COVID-19 confirmed cases and deaths are reported in [21].

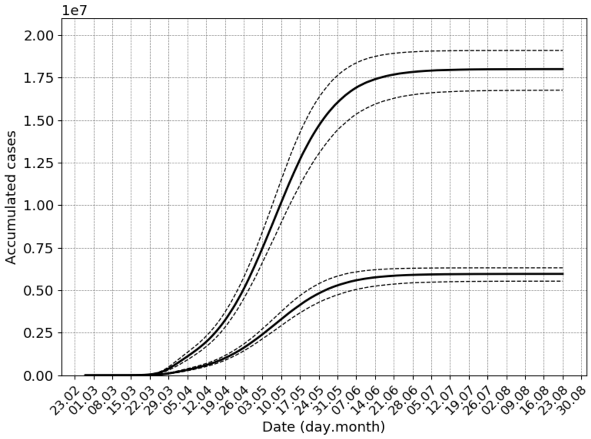

Fig S9. Outbreak analysis for Mexico city metropolitan area, data until 15 May. Total number of recovered $R(\infty)$ with $N_{eff} = N$ (the total population) and $f = 0.05$ reaches approximately 17.5×10^6. In the case $N_{eff} = N/3, f = 0.15$, the recovered $R(\infty)$ only reaches roughly 5.5×10^6. However, the fit for cases and deaths and the predictive curves for hospital demand are identical (results not shown).

References

1. GREEN PJ. Reversible jump Markov chain Monte Carlo computation and Bayesian model determination. Biometrika. 1995;82(4):711–732. doi:10.1093/biomet/82.4.711.

2. Cori A, Ferguson NM, Fraser C, Cauchemez S. A New Framework and Software to Estimate Time-Varying Reproduction Numbers During Epidemics. American Journal of Epidemiology. 2013;178(9):1505–1512. doi:10.1093/aje/kwt133.

3. Spiegelhalter DJ, Best NG, Carlin BP, Van Der Linde A. Bayesian measures of model complexity and fit. Journal of the royal statistical society: Series b (statistical methodology). 2002;64(4):583–639.

4. Van den Driessche P, Watmough J. Reproduction numbers and sub-threshold endemic equilibria for compartmental models of disease transmission. Mathematical biosciences. 2002;180(1-2):29–48.

5. Ferguson NM, Laydon D, Nedjati-Gilani G, Imai N, Ainslie K, Baguelin M, et al. Impact of non-pharmaceutical interventions (NPIs) to reduce COVID-19 mortality and healthcare demand. London: Imperial College COVID-19 Response Team, March. 2020;16.

6. Champredon D, Dushoff J, Earn DJ. Equivalence of the Erlang-distributed SEIR epidemic model and the renewal equation. SIAM Journal on Applied Mathematics. 2018;78(6):3258–3278.

7. Verity R, Okell LC, Dorigatti I, Winskill P, Whittaker C, Imai N, et al. Estimates of the severity of COVID-19 disease. medRxiv. 2020;.

8. Team EE, et al. Updated rapid risk assessment from ECDC on the novel coronavirus disease 2019 (COVID-19) pandemic: increased transmission in the EU/EEA and the UK. Eurosurveillance. 2020;25(10).

9. Zhang J, Litvinova M, Wang W, Wang Y, Deng X, Chen X, et al. Evolving epidemiology and transmission dynamics of coronavirus disease 2019 outside Hubei province, China: a descriptive and modelling study. The Lancet Infectious Diseases. 2020;.

10. Buchholz U, et al.. Modellierung von Beispielszenarien der SARS-CoV-2-Ausbreitung und Schwere in Deutschland; 2020.

11. Novel CPERE, et al. The epidemiological characteristics of an outbreak of 2019 novel coronavirus diseases (COVID-19) in China. Zhonghua liu xing bing xue za zhi= Zhonghua liuxingbingxue zazhi. 2020;41(2):145.

12. Zou L, Ruan F, Huang M, Liang L, Huang H, Hong Z, et al. SARS-CoV-2 viral load in upper respiratory specimens of infected patients. New England Journal of Medicine. 2020;382(12):1177–1179.

13. He X, Lau EH, Wu P, Deng X, Wang J, Hao X, et al. Temporal dynamics in viral shedding and transmissibility of COVID-19. Nature medicine. 2020; p. 1–4.

14. Lindén A, Mäntyniemi S. Using the negative binomial distribution to model overdispersion in ecological count data. Ecology. 2011;92(7):1414–1421.

15. Christen JA, Fox C. A general purpose sampling algorithm for continuous distributions (the t -walk). Bayesian Anal. 2010;5(2):263–281. doi:10.1214/10-BA603.

16. Geyer CJ. Practical markov chain monte carlo. Statistical science. 1992; p. 473–483.

17. Park SW, Bolker BM, Champredon D, Earn DJ, Li M, Weitz JS, et al. Reconciling early-outbreak estimates of the basic reproductive number and its uncertainty: framework and applications to the novel coronavirus (SARS-CoV-2) outbreak. medRxiv. 2020;.

18. Singh VP, Rajagopal A, Singh K. Derivation of some frequency distributions using the principle of maximum entropy (POME). Advances in Water Resources. 1986;9(2):91–106.

19. SEDATU-CONAPO-INEGI. Delimitación de las zonas metropolitanas de México 2015; 2020. Available from: `https://www.gob.mx/conapo/documentos/delimitacion-de-las-zonas-metropolitanas-de-mexico-2015.html`.

20. CONAPO. Proyecciones de la Población de México y de las Entidades Federativas, 2016-2050; 2020. Available from: `https://datos.gob.mx/busca/dataset/proyecciones-de-la-poblacion-de-mexico-y-de-las-entidades-federativas-201`

21. de Salud S. Datos Abiertos - Dirección General de Epidemiología; 2020. Available from: `https://www.gob.mx/salud/documentos/datos-abiertos-152127`.
